# Magnetic properties and magnetocrystalline anisotropy of Nd_2_Fe_17_, Nd_2_Fe_17_X_3_, and related compounds

**DOI:** 10.1038/s41598-018-21969-8

**Published:** 2018-02-26

**Authors:** Tribhuwan Pandey, David S. Parker

**Affiliations:** Material Science and Technology Division, Oak Ridge National Laboratory, Oak Ridge, Tennessee, 37831 USA

## Abstract

The electronic and magnetic properties of Nd_2_Fe_17_ and Nd_2_Fe_17_X_3_ (X = C or N) compounds have been calculated using the first-principles density functional calculations. Among these, the nitrogen and carbon interstitial compounds exhibit all of the required properties such as a saturation moment of 1.6 T, Curie temperature of 700–750 K, however easy magnetic axis lies in the planar direction making them less attractive for permanent magnet applications. The calculated magnetocrystalline anisotropy energy is found to be −2.7 MJ/m^3^ for Nd_2_Fe_17_C_3_ and −4.7 MJ/m^3^ for Nd_2_Fe_17_N_3_. We further explored the possibility of changing the easy axis direction through La/Ce alloying at Nd site. Although the MAE is found to be smaller in magnitude for all the La/Ce alloys it still maintains planar direction.

## Introduction

The Magnetocrystalline anisotropy energy (MAE), the energy required to switch crystal magnetization from the favorable direction to other spatial directions is the most important property of a ferromagnet. As formulated by Van Vleck^1^, in general MAE is an intrinsic property which mainly originates from spin-orbit coupling (SOC) interaction. A large value of MAE is essential for wide range of applications, from permanent magnets to magnetic storage devices^2–8^. The SOC is strong in heavy elements such as rare-earth compounds which consequently exhibit large MAE. Together with large MAE a good permanent magnet requires large value of magnetization, large coercivity, high temperature stability and high Curie temperature^6,7^. For example, one of the best magnetic materials, Nd_2_Fe_14_B, has T_*c*_ 588 K, M_*s*_ 1.28 MA/m, K_1_ 4.9 MJ/m^3^ and an easy magnetic axis along the *c*-axis^9^.

Nd_2_Fe_17_ is another Nd based interesting magnetic material. Nd_2_Fe_17_ is known to crystallize in rhombohedral Th_2_Zn_17_ structure type with the space group *R*−3 *m*. This compound has gained back interest since it was shown that insertion of interstitial atoms could raise the usually low Curie temperature (T_*c*_) of this ferromagnetic compound. In the case of Nd_2_Fe_17_, carbon and nitrogen insertion increases T_*c*_ from 325 K to 658 K for carbon interstitial (Nd_2_Fe_17_C_2_) and to 732 K for nitrogen interstitial (Nd_2_Fe_17_N_2.3_) compound^10^. Although both carbon and nitrogen interstitial compounds exhibit high magnetic moments and Curie point^11–15^, unlike Sm_2_Fe_17_C_3_(N_3_)^8,11–14,16–19^ they suffer from planar MAE. One possibility for changing the easy axis direction is through alloying. Previous theoretical calculations^20,21^ for these compounds treat Nd *f* electrons as core electrons (also known as open core approximation) which do not take part in hybridization. However for the rare earth elements the most of the contribution to MAE is shown to be from *f* electrons, hence a proper treatment of *f* electrons is crucial for accurate description of MAE^22–24^. To this end, here we have applied a Hubbard U correction on the Nd-*f* orbitals which splits the *f* bands into lower and upper Hubbard bands. Our results are in better agreement with experiments than previous theoretical studies where *f* orbitals were treated under open core approximation rather than as valence electrons. We also investigate the effect of elemental substitutions of La and Ce on Nd site on MAE. Our calculations show these substitutions do not switch the easy axis direction.

## Results

**Figure 1:**
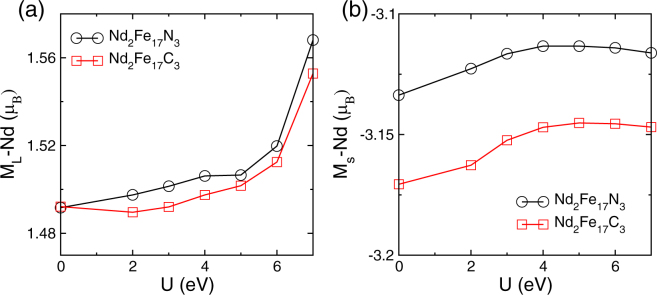
U dependence of calculated (**a**) Nd orbital and (**b**) spin magnetic moments for Nd_2_Fe_17_N_3_, and Nd_2_Fe_17_C_3_.

We begin our study by determining the ground state for Nd_2_Fe_17_ and its carbides and nitrides. To estimate the ground state, calculations were performed by assuming, non magnetic, ferromagnetic (parallel alignment of spins between Fe and Nd atoms), and anti-ferromagnetic (anti-parallel alignment of spins between Fe and Nd atoms) configurations. For all three systems Nd_2_Fe_17_, Nd_2_Fe_17_C_3_, and Nd_2_Fe_17_N_3_ we find the ground state to have the Nd spin moment opposite to that of the Fe and energy difference between ground state and non magnetic was found to be as 360, 460, and 510 meV on per Fe atom basis. The calculated magnetic moments are summarized in Table 1. The variation in Nd orbital and spin magnetic moments for Nd_2_Fe_17_N_3_ and Nd_2_Fe_17_C_3_ is presented in Fig. 1(a) and (b) respectively. As can be seen from Fig. 1(a) the orbital magnetic moments (M_*L*_) is positive for rare earth atom, indicating that the direction of the orbital magnetic moment is opposite to the spin magnetic moment. This is consistent with the Hund’s rule for half filled rare earth ions. The calculated orbital moment of Nd atoms without U is 1.49 *μ*_*B*_ for both Nd_2_Fe_17_N_3_ and Nd_2_Fe_17_C_3_. On varying U in GGA + SOC + U calculations, although the orbital moment of Nd atoms increases, overall the orbital moment displays a weak dependence on U. The rate of increase of orbital moments with U is higher for carbon interstitial compound than in nitrogen interstitial compound. The variation of Nd spin moments with U parameter is also shown in the Fig. 1(b). The Nd spin moments are almost independent of U values used.

**Table 1:** Calculated total (orbital + spin) magnetic moments at various atom sites in *μ*_B_, total magnetic moment of the system (m_*tot*_) in *μ*_B_ per formula unit, and magnetocrystalline anisotropy constant (K_1_) in MJ/m^3^, for Nd_2_Fe_17_, Nd_2_Fe_17_X_3_, NdLaFe_17_X_3_, and NdCeFe_17_X_3_. Here X represents carbon or nitrogen atoms. These values are calculated including spin orbital coupling (SOC) along with a U value of 3 eV and 5 eV at Ce and Nd sites, respectively. For comparison experimental total magnetization (m_tot_ (exp) in *μ*_B_ per formula unit) and MAE (K_1_(exp) in MJ/m^3^) values are also shown.

	Nd_2_Fe_17_	Nd_2_Fe_17_C_3_	Nd_2_Fe_17_N_3_	NdLaFe_17_C_3_	NdCeFe_17_C_3_	NdLaFe_17_N_3_	NdCeFe_17_N_3_
X(9*e*)		−0.15	−0.04	−0.15	−0.13	−0.04	−0.04
Fe(9*d*)	1.61	2.65	2.65	2.64	2.62	2.64	2.65
Fe(18*f*)	2.32	1.93	2.20	1.92	1.94	2.19	2.21
Fe(18*h*)	2.26	2.18	2.37	2.17	2.18	2.36	2.37
Fe(6*c*)	2.48	2.47	2.47	2.46	2.47	2.45	2.48
RE(6*c*)	−1.60	−1.61	−1.61	−1.63	−1.60	−1.62	−1.63
La/Ce				−0.15	−0.23	−0.11	−0.24
K_1_	−2.1	−2.7	−4.7	−1.06	−1.6	−3.2	−3.5
K_1_(exp)	−3.7^12^		−6.8^8,11^				
m_*tot*_	33.03	33.0	36.3	34.4	35.0	37.7	38.0
m_*tot*_ (exp)	36.6^12^		40.2^19^

**Figure 2:**
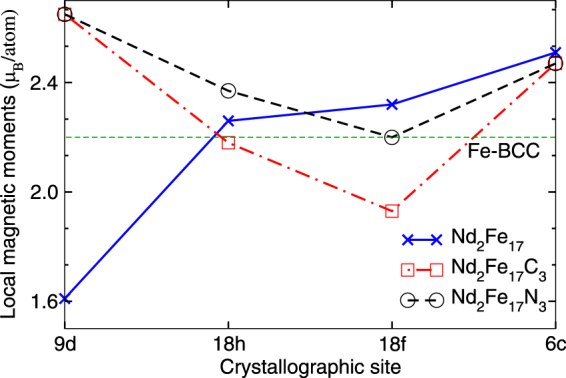
Calculated local magnetic moments for the crystallographically nonequivalent Fe sites in Nd_2_Fe_17_ (blue cross), Nd_2_Fe_17_C_3_ (red squares), and Nd_2_Fe_17_N_3_ (black circles). The calculated magnetic moments for BCC-Fe is also shown for comparison.

The calculated Fe orbital moments for Nd_2_Fe_17_ lie between 0.04 to 0.05 *μ*_*B*_. On introducing interstitial nitrogen/carbon atom the Fe orbital moments are increased, and the enhancement is higher for Nd_2_Fe_17_C_3_. The total magnetic moments for the crystallographically nonequivalent Fe sites are plotted in Fig. 2. The calculated total magnetic moments agree reasonably with experiments. Furthermore, on introducing nitrogen/carbon interstitial atoms, while the spin moment of Fe-18*f* and Fe-18*h* sites, which are close to N/C atoms decreases, the moments on the distant Fe-9*d* site are enhanced as shown in Fig. 2 and Table 1. This trend of Fe moments on nitrogenation/carbonation is in good qualitative agreement with previous studies and has been attributed to hybridization between N(C) and Fe atoms^21^. Similar variation in magnetic moments upon nitrogenation and carbonization has also been observed for Sm_2_Fe_17_. Regardless of this slight variation the average Fe-spin moment of these systems remains in the range of 2.3–2.5 *μ*_*B*_, which is significantly higher than the value for BCC Fe 2.2 *μ*_*B*_. The experimental values of Fe moments for Nd_2_Fe_17_N_3_ by neutron powder diffraction measurements show large variation depending on the stoichiometry of the nitrogen in the system. For example the total magnetic moments of Nd_2_Fe_17_N_2.85_ and Nd_2_Fe_17_N_2.91_ are 32.5 *μ*_*B*_ and 40.6 *μ*_*B*_, respectively^19^, indicating the sensitivity of magnetic properties on nitrogen stoichiometry. This large variation is mainly due to the difference in Fe-Fe distance depending upon on nitrogen stoichiometry. As explained above due to the sensitivity of magnetic properties on nitrogen/carbon content, a good quantitative agreement between experiments and theory is unlikely. Nonetheless our calculated total magnetic moment of 36.3 *μ*_*B*_ for Nd_2_Fe_17_N_3_ lies well within the range proposed by experimental measurements as shown in Table 1. The calculated Nd total magnetic moments are also listed in Table 1. We see that due to the presence of 4*f* electrons, Nd atom has a large (~1.47 *μ*_*B*_) orbital moment. For both carbon and nitrogen interstitial compounds the orbital moment of Nd is slightly increased from 1.47 *μ*_*B*_ to 1.50 *μ*_*B*_. The magnetic moment for La/Ce substituted compounds are also listed in Table 1. For the substituted compounds the magnetic moment on Fe and Nd sites mostly remain unchanged. On the other hand the magnetic moment on the substituted RE atomic sites (La/Ce) is greatly reduced as La has no *f* electrons and Ce has only one outermost *f* electron.

**Figure 3:**
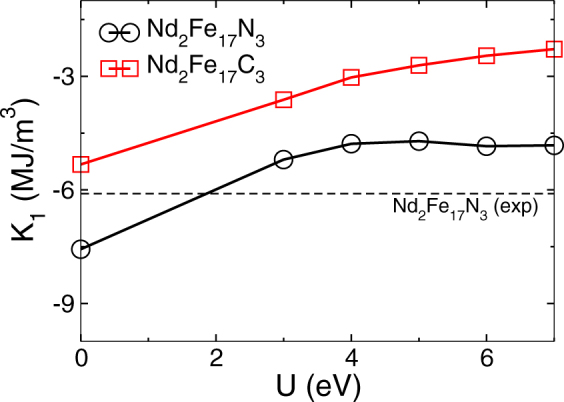
The Hubbard U dependence of calculated magneto-crystalline anisotropy energy (MAE) for Nd_2_Fe_17_N_3_, and Nd_2_Fe_17_C_3_. Experimental value of MAE for Nd_2_Fe_17_N_3_ is also marked for comparison.

Next, we calculate the magnetocrystalline anisotropy energy (MAE) constant K_1_, which is shown in Table 1. MAE is an important parameter for generating high coercivity in a permanent magnets. For accurate estimate of MAE values a proper treatment of strongly correlated *f* electrons is essential by applying a onsite Hubbard U parameter. Here a U value of 3 and 5 eV is used for Ce, and Nd, respectively. The convergence of MAE with respect to U parameter is shown in Fig. 3. For Nd_2_Fe_17_N_3_ where the experimental data of MAE is available, our calculated MAE with U_Nd_ as 5 eV is in reasonable agreement with the experimental data. We found the parent compound Nd_2_Fe_17_ to be planar with K_1_ value as −2.1 MJ/m^3^, which is in fair agreement with the experimentally measured value of −3.7 MJ/m^3^^12^. Unlike Sm_2_Fe_17_ where carbon and nitrogen interstitial atoms switch the direction of MAE from planar to axial, Nd_2_Fe_17_N_3_ and Nd_2_Fe_17_C_3_ does not exhibit this change in MAE sign and still maintain planar anisotropy. This planar anisotropy is less desirable for permanent magnet applications. However the magnitude of K_1_ is quite significant and a value of −2.7 MJ/m^3^ and −4.7 MJ/m^3^ was obtained for Nd_2_Fe_17_C_3_ and Nd_2_Fe_17_N_3_, respectively. The measured anisotropy values for Nd_2_Fe_17_N_3_ are also listed in Table 1, which is in fair agreement with our calculated value. Given the high magnetization along with large magnitude of K_1_ one might obtain a coercivity higher than 1.5 Tesla in these compounds if the MAE was uniaxial.

To explore the possibility of switching the sign of MAE and reducing the rare earth content in these magnets next we study the effect of La/Ce substitution on MAE. As the MAE is sensitive to the nature of electronic structure around the Fermi energy, it can be controlled by tuning the band structure around the Fermi energy. In practice this can be done, for example, by doping/alloying. Theoretical calculations were done in order to find a possible alloy based on NdLaFe_17_X_3_ and NdCeFe_17_X_3_ (X = C or N) with uniaxial anisotropy. We thus began with substituting one out of two Nd atoms in the primitive cell by La/Ce. Results of these calculations are summarized the in Table 1. As seen, for all the La/Ce substituted systems the MAE still remains planar. While for Ce substituted compounds the MAE is reduced by 25–31% (on going from nitrogen interstitial to carbon interstitial), a higher 40–60% reduction was observed for La substituted compounds.

**Figure 4:**
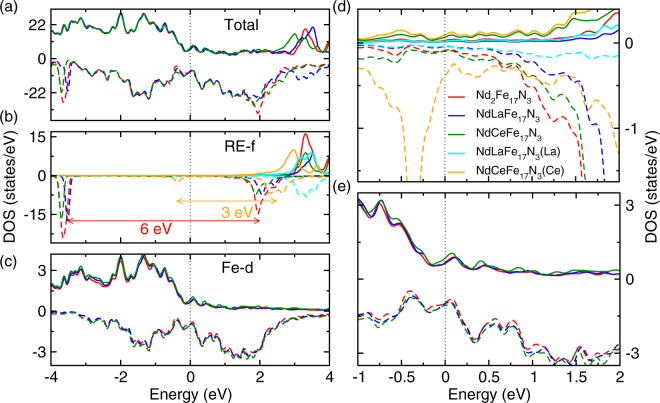
The total and partial density of states for Nd_2_Fe_17_N_3_ (red), NdLaFe_17_N_3_ (blue), NdCeFe_17_N_3_ (green), La in NdLaFe_17_N_3_ (cyan), and Ce in NdCeFe_17_N_3_ (orange). In panel (a) the Total DOS (b) the RE atom DOS, and (c) the averaged Fe atoms DOS are compared. Panel (d) and (f) show the zoomed version of panel (b) and (c) in the vicinity of Fermi level. The positive and negative DOS values correspond to spin-up and spin-down channels respectively.

Figure 4 presents the density of states (DOS) for Nd_2_Fe_17_N_3_. The total DOS illustrates behavior of a ferromagnetic system. In the Fig. 4(b) and (c) the DOS for RE-4*f* and Fe-3*d* states are shown. On comparing the total DOS with partial DOS in the lower panels, we can see that the states in the vicinity of the Fermi level are mainly composed of Fe-3*d* states. The RE-4*f* states are not occupied in the spin-up channel and are partially occupied in the spin-down channel, confirming that the Nd spin-moments align in the opposite direction compared to the Fe moments. Due to Hubbard U parameter the Nd spin down band split into lower and upper Hubbard bands separated by 6 eV (marked by red arrow in Fig. 4(b)). To understand the effect of La/Ce substitution at the Nd site on the magnetic properties of these compounds, we next analyze their DOS. The total and partial DOS for the these compounds are shown in Figs. 4(a)–(c). The zoomed version of RE-*f* DOS and Fe-*d* DOS are shown in Figs. 4(d) and (e). As shown in Figs. 4(c) and (e) Ce/La substitution at Nd site does not affect Fe-*d* DOS which overlaps with the Fe-*d* DOS for Nd_3_Fe_17_N_3_. Due to this reason the magnetic moments on Fe sites are largely unaffected by La/Ce substitution. The only significant change is in the RE-*f* states, which are shifted to higher energies. As shown in Fig. 4(d) upon La/Ce substitution the Nd DOS around Fermi level is reduced. This reduction in RE DOS is responsible for the reduction in magnitude of MAE on La/Ce substitution (Table 1).

**Figure 5:**
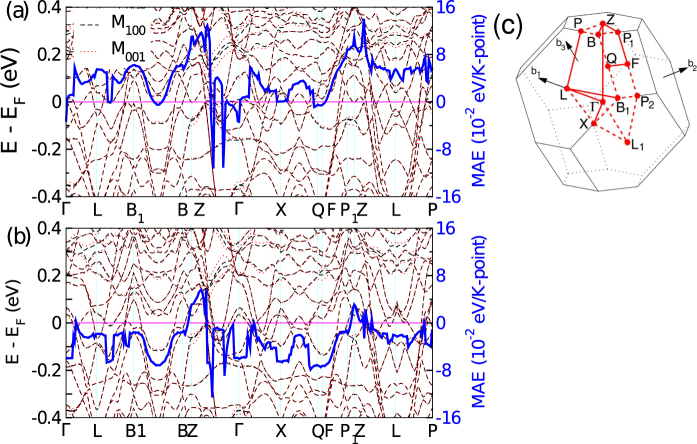
Band structure including SOC with magnetization along inplane [11-2] direction (marked as M_100_ by black dash-dotted line) and out of plane [111] direction (marked as M_001_ by red dashed line) as well as the MAE contribution per ***k***-point (blue solid line), calculated via the magnetic force theorem (**a**) Sm_2_Fe_17_N_3_ and (**b**) Nd_2_Fe_17_N_3_. The band energies are marked on the left *y*-axis and MAE contribution per *k*-points is marked on the right *y*-axis. In order to capture the MAE behavior at various band crossing, band structure was plotted by taking 3000 ***k***-points along the entire high symmetric path. (**c**) The Brillouin along with the high symmetry directions.

The important insights into the effect of SOC on magnetic properties can be obtained from perturbation theory^25–27^. According to this theory the MAE can be described by the electronic structure near the Fermi energy, in-terms of coupling between occupied and unoccupied levels. To obtain information about which regions are particularly important to the MAE, the band structures after applying SOC with magnetization along either in plane or out of plane directions are plotted in Fig. 5(a) and (b)). From these bands the MAE contribution per ***k***-point can be evaluated using the magnetic force theorem^28–30^, by taking the difference of the sum over occupied energy eigenvalues for different magnetization directions, which is also plotted (blue line, right *y*-axis) in Fig. 5(a) and (b) for Sm_2_Fe_17_N_3_ and Nd_2_Fe_17_N_3_ respectively. The force theorem calculations produce a MAE of 12.1 MJ/m^3^ for Sm_2_Fe_17_N_3_, and −4.1 MJ/m^3^ for Nd_2_Fe_17_N_3_, which is in nice agreement with the values obtained from total energy calculations. Since the total MAE is positive for Sm_2_Fe_17_N_3_, as can be seen in Fig. 5(a) overall the MAE remains positive throughout the Brillouin zone (except along Γ to $\frac{1}{2}-Z$ direction where it takes a negative value). On the other hand for Nd_2_Fe_17_N_3_ although the overall profile of MAE with respect to ***k***-points remains the similar as Sm_2_Fe_17_N_3_, MAE takes negative values in the majority of brillouin zone.

**Figure 6:**
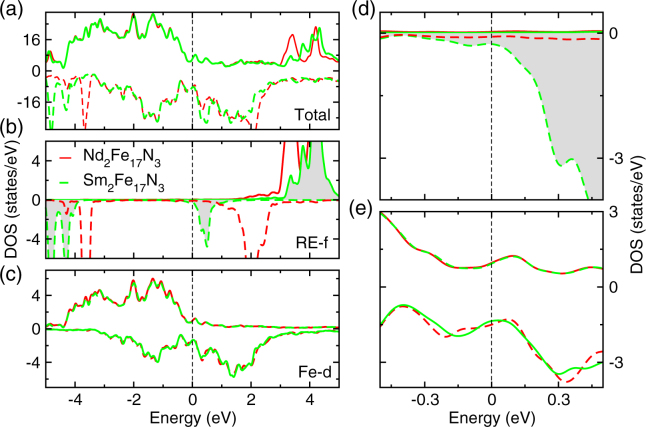
Comparison of density of states for Nd_2_Fe_17_N_3_ and Sm_2_Fe_17_N_3_. The DOS is plotted within GGA + SOC + U calculations with a U value of 5 eV for Nd and Sm sites. In panel (a) the Total DOS (b) the RE atom DOS, and (c) the averaged Fe atoms DOS are compared. Panel (d) and (f) show the zoomed version of panel (b) and (c) in the vicinity of Fermi level.

Figure 6 compares the DOS for Nd_2_Fe_17_N_3_, and Sm_2_Fe_17_N_3_. As shown in the Fig. 6(a) in close vicinity of Fermi level (*E*_F_), the majority spin DOS is similar for the two compounds. However, as Sm is exchanged for Nd more electrons are added into the system and the minority spin states become occupied, whereby these 4*f* levels are shifted more towards the left in the middle panel of Fig. 6(b) and, as a result, Sm-4*f* level in the minority spin DOS of Sm_2_Fe_17_N_3_ shifts towards Fermi level. The Fe-DOS for both majority and minority spin in both Nd_2_Fe_17_N_3_, and Sm_2_Fe_17_N_3_ is quite similar. The important observation is the presence of minority 4*f* spin channels at the the Fermi level of large uniaxial anisotropy in Sm_2_Fe_17_N_3_, although other differences in the electronic structure are also important.

**Figure 7:**
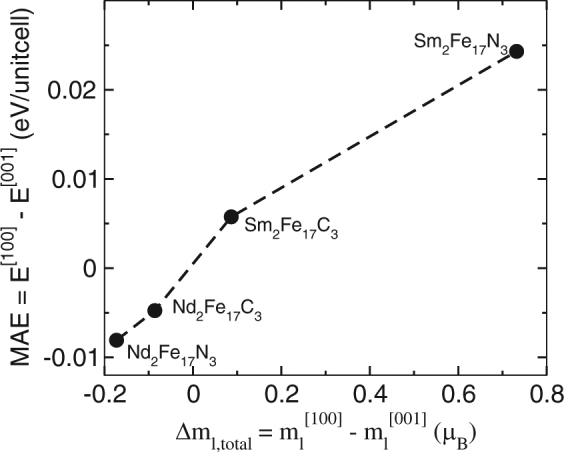
Calculated MAE vs orbital magnetic anisotropy for Nd_2_Fe_17_C_3_, Nd_2_Fe_17_N_3_, Sm_2_Fe_17_C_3_, and Sm_2_Fe_17_N_3_. The orbital magnetic anisotropy is averaged over both Nd/Sm and Fe atoms.

The MAE of a permanent magnet is dependent on SOC, and can be further quantified by considering its dependence on orbital moments. As shown by Bruno *et al*.^26^ by using perturbation theory that if deformations of the Fermi surface can be neglected and the MAE is dominated by spin-diagonal coupling, the MAE and orbital magnetic-moment anisotropy are proportional. The relation between MAE and orbital magnetic anisotropy is presented in Fig. 7. As can be seen from Fig. 7 the MAE is directly proportional to the orbital magnetic anisotropy. For Sm_2_Fe_17_C_3_, and Sm_2_Fe_17_N_3_ where a uniaxial anisotropy is obtained the orbital magnetic moments is also maximum along easy axis. On the other for Nd_2_Fe_17_C_3_, and Nd_2_Fe_17_N_3_ where the anisotropy is in hexagonal plane, orbital magnetic moment is maximum along the hard magnetization axis.

## Discussions

We present a computational study of intrinsic magnetic properties such as saturation magnetization and MAE for Nd_2_Fe_17_, Nd_2_Fe_17_X_3_ and related compounds. For all compounds the rare earth and Fe atoms spin moments are anti-aligned in the ground state. The calculated magnetic properties of the base compounds agree well with available experimental data. The treatment of the Nd-*f* electrons in our calculations is different from the previously reported first principles calculations. For example the calculation of Lai *et al*.^15^ which were performed within orthogonalized linear combination of atomic orbitals (OLCAO) method, reported a decrease in total magnetic moments from 37.3 *μ*_*B*_/unit-cell (for to Nd_2_Fe_17_) to 36.3 *μ*_*B*_/unit-cell (for Nd_2_Fe_17_N_3_) which is contradictory to the experimental observation^19^, where on nitrogenation an increase in total magnetic moment has been reported. As shown in Table 1 the observed enhancement of magnetic moments in Nd_2_Fe_17_ upon nitrogenation (for Nd_2_Fe_17_N_3_) is nicely captured by our GGA + SOC + U calculations. Similarity Drebov *et al*.^20^ used open-core method for treatment of *f*-electrons by assuming RE^3+^ configuration. Such treatment of *f* electrons cannot reproduce some of the observed magnetic properties. For instance within open core method a moment of 2.5 *μ*_*B*_ has been reported on Nd site, which is overestimated on comparing with the available experimental data^19^ (1.6 *μ*_*B*_). On the other hand, within our calculations the *f*-electrons are treated as valence electrons and, as a result we could correctly reproduce the reported moment for Nd site. Overall our all-electron calculations can reproduce a number of experimentally observed properties. For example, experimentally^19^ for Nd_2_Fe_17_ Fe-9*d* site has the lowest magnetic moment of 1.6 *μ*_*B*_. Upon introducing the interstitial nitrogen the moment of Fe-9*d* increases significantly and for Nd_2_Fe_17_N_3_ the Fe-9*d* site has the maximum moment. This is nicely produced by our calculations as shown in Fig. 2. Furthermore the calculated MAE for Nd_2_Fe_17_ and Nd_2_Fe_17_N_3_ is in fair agreement with the available experimental data as shown in Table 1.

Although Nd_2_Fe_17_X_3_ compounds has many of the desirable properties for a permanent magnet such as high Curie point, large saturation magnetization, the easy axis of magnetization lies in the hexagonal plane hindering it practical application. To investigate the possibility of switching the MAE direction from hexagonal plane to *c*-axis we also investigated the effect of La/Ce substitution at Nd site. These calculations indicate that although the MAE on La/Ce substitution is reduced by 30–40%, its still lies in the hexagonal plane, which is not very promising for technological applications. The MAE has been also analyzed in terms of the electronic structure and by using the magnetic force theorem to compute k-point resolved contributions to the MAE. For Nd_2_Fe_17_N_3_ the density of states at the Fermi energy is dominated by 3*d* states, 4*f* states only contribute notably to the MAE in small regions of the Brillouin zone, however for Sm_2_Fe_17_N_3_ the DOS around the Fermi level is dominated by both Fe-3*d*, Sm-4*f* states. As the Fe-*d* states exhibit similar features for Nd_2_Fe_17_N_3_ and Sm_2_Fe_17_N_3_, the origin of uniaxial MAE could lie in the 4*f*-3*d* hybridization.

## Methods

Density functional theory (DFT) calculations in the generalized gradient approximation^31^ (GGA) were performed with the full-potential linearized augmented plane waves (FP-LAPW) method as implemented in WIEN2k^32–34^. The sphere radii were set to 2.50, 1.88, 1.61, and 1.61 Bohr for Nd, Fe, N and C. All calculations were performed with the experimental lattice parameters^8,35,36^. The structure relaxations were performed within spin-polarized calculations without spin orbit coupling and all internal coordinates were relaxed until internal forces on atoms were less than 1 mRyd/Bohr. For structural relaxation 500 **k**-points were in the full Brillouin zone. SOC was included in the MAE calculations within a second variational approach^37^. For all the calculations a *RK*_max_ = 7 was used. *RK*_max_ is typically defined as the product of the smallest muffin-tin sphere and the largest reciprocal lattice vector, and describes the basis set size for a calculation. For La/Ce substitution at Nd site, one out of two Nd atoms in the primitive cell was replace by La/Ce. These substituted structure were subsequently relaxed to their ground state by minimizing the forces on all the atoms.

All the calculations are performed in the collinear spin alignment. The magnetic anisotropy energy (MAE) is obtained by calculating the total energies of the system with spin obit coupling (SOC) as K = E_*a*_ − E_*c*_, where E_*a*_ and E_*c*_ are the total energies for the magnetization oriented along the *a* and *c* directions, respectively. Positive (negative) K corresponds to uniaxial (planar) anisotropy. For MAE calculations the convergence with respect to K-points was carefully checked all the MAE results reported in this paper correspond to 2000 reducible K-points in full Brillouin zone. To correctly treat the strong interactions between the Nd/Ce-*f* electrons, the Hubbard “U” correction was applied with U_*Nd*_ = 5.0 eV, and U_*Ce*_ = 3.0 eV with the Hund’s coupling parameter J as zero. For DFT + U calculations, the standard self interaction correction (SIC) method^38,39^ was used where onsite Coulomb interaction for localized orbitals is parametrized by U_effective_ = U − J. The values of U parameter were obtained by optimized various magnetic properties with respect to available experimental data, and lies in the typical range that has been used successfully to describe various properties of Nd/Ce compounds before^22,40–43^.
